# Hormesis of Glyceollin I, an Induced Phytoalexin from Soybean, on Budding Yeast Chronological Lifespan Extension

**DOI:** 10.3390/molecules19010568

**Published:** 2014-01-06

**Authors:** Yuancai Liu, Ziyun Wu, Shengbao Feng, Xuena Yang, Dejian Huang

**Affiliations:** 1 Hubei Key Laboratory of TCM Based Functional Food Quality and Safety, Jing Brand Company, Daye 435100, Hubei, China; E-Mails: lyc@jingpai.com (Y.L.); fsb@jingpai.com (S.F.); 2 National University of Singapore (Suzhou) Research Institute, 377 Lin Quan Street, Suzhou Industrial Park, Jiangsu 215123, China; E-Mail: chmhdj@nus.edu.sg; 3 Food Science and Technology Program, Department of Chemistry, National University of Singapore, 3 Science Dr. 3, Singapore 117543, Singapore; 4 College of Bioscience & Biotechnology, Fuzhou University, Fuzhou 350108, China; E-Mail: yangxna@hotmail.com

**Keywords:** chronological life span, glyceollin I, hormesis, induced phytoalexin, aging

## Abstract

Glyceollin I, an induced phytoalexin isolated from soybean, has been reported to have various bioactivities, including anti-bacterial, anti-nematode, anti-fungal, anti-estrogenic and anti-cancer, anti-oxidant, anti-inflammatory, insulin sensitivity enhancing, and attenuation of vascular contractions. Here we show that glyceollin I has hormesis and extends yeast life span at low (nM) doses in a calorie restriction (CR)-dependent manner, while it reduces life span and inhibits yeast cell proliferation at higher (μM) doses. In contrast, the other two isomers (glyceollin II and III) cannot extend yeast life span and only show life span reduction and antiproliferation at higher doses. Our results in anti-aging activity indicate that glyceollin I might be a promising calorie restriction mimetic candidate, and the high content of glyceollins could improve the bioactivity of soybean as functional food ingredients.

## 1. Introduction

Legumes have high nutritional value and play an important role in traditional diets throughout the world. Recent studies have suggested that legumes, especially soybean and peanuts, could be good functional foods for health promotion [1]. Legume seed sprouts are also popular foods globally. During germination, some components of the seed are degraded and used for respiration and synthesis of new cell constituents for the plant development, which causes significant changes in the biochemical characteristics. For example, they produce secondary metabolites to enhance their stress resistance [2]. Plants possess both constitutive and inducible mechanisms to resist stress from wounds, freezing, ultraviolet light, and microorganisms (e.g., oomycetes, fungi, bacteria, viruses, and insects). In some cases, phytoalexins are induced in plants for self-defense against microbial infections. Up to now, a large number of phytoalexins have been isolated and identified [3]. Their functions in plant mainly include antimicrobial and antioxidant activities [4,5].

Some food grade microorganisms have been used as starters in fermentation processes such as *Rhizopus oligosporus* for tempeh fermentation and *Bacillus subtilis* in natto fermentation. They are attractive stressors that induce phytoalexins from legume seeds. Our previous work has shown that food grade microbial-stressed (*R. oligosporus*) germination of soybeans leads to generation of a group of oxooctadecadienoic acids and their glyceryl esters in addition to glyceollins, known phytoalexins presented in stressed soybeans. In addition, the nutritional values of the soybean foods made from the bean seeds may be particularly beneficial, with higher contents of total isoflavones [6]. Furthermore, the food grade fungal stress on germinating peanut seeds can induce a number of stilbenoid phytoalexins and enhance polyphenolic antioxidants [7,8]. Resveratrol is a well-studied stilbenoid phytoalexin that has received tremendous attention because of its broad range of health benefits in a variety of human disease models, including cardio- and neuro-protection, immune regulation, and cancer chemoprevention [9]. Logically, the introduction of phytoalexins in food through bioprocesses is an emerging field of functional food research [5].

Natural products with anti-aging activity have been receiving great attention in the academic community. Calorie restriction (CR), the reduction of nutrient intake without malnutrition [10], is a gold standard method in aging research and is still the only dietary intervention shown to extend the average and maximum life span in model organisms from yeast to primates [11]. If life span extension by CR functions through conserved mechanisms, using model organisms to interpret the genetic pathways involved may allow for the design and screening of target molecules as CR mimetics, without the need for strict dietary regimes and the associated detrimental side effects, physical or psychological, that CR can impose [11]. So far a few natural products such as resveratrol [12,13] and rapamycin [14] have been shown to be calorie restriction mimetics (CRM) in several organisms [15]. Resveratrol can extend life span in budding yeast *Saccharomyces cerevisiae* (replicative life span), *Caenorhabditis elegans* and *Drosophila melanogaster*, but not in mice [16]. Rapamycin, isolated from the bacterium *Streptomyces hygroscopicus*, has potent immunosuppressive and antiproliferative properties, while it can extend median and maximal life span of mice, even when they are fed at 20 months of age (equivalent to 60 human years) [14].

Hormesis is an adaptive response of cells or organisms to a moderate stress. It describes the dose-response relationship of stressors (e.g., chemical, thermal, or radiological) that are noxious at higher levels but can exert a beneficial effect on cells at low doses by inducing a response that results in enhanced stress resistance [17,18]. Rapamycin and resveratrol are antifungal natural products at high concentrations and can induce defense responses at low doses in fungi, nematodes, flies, fish, and mice [19]. CR is probably one of the most well recognized hormetic phenomena capable of increasing mammalian life span. These stressors, in large part via activation of conserved stress-response signal transduction pathways, decrease risks of common age-related conditions, such as cancer, cardiovascular diseases, type 2 diabetes, and neurological diseases, and hence lengthening the life span [20].

Recently, we have developed a high throughput assay to determine yeast chronological life span (CLS) [21]. Interestingly, after screening a number of natural products, we found that an induced phytoalexin, glyceollin I, could similarly function as a hormesis agent in yeast and extend its life span through a CR-dependent regime at low doses.

## 2. Results and Discussion

### 2.1. Antiproliferation Activity of Glyceollins

**Figure 1:**
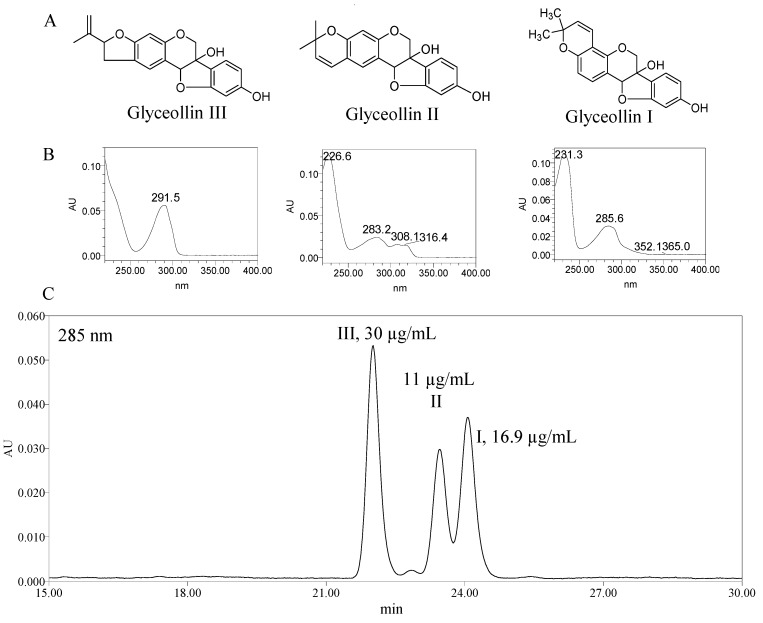
Structure (**A**), UV absorption spectra (**B**), and HPLC chromatogram at 285 nm (**C**) of glyceollin isomers I, II, and III obtained from black soybean sprouts with food grade fungus *R. oligosporus* stress.

The glyceollins I, II, and III were isolated by silica gel column chromatography (Figure 1) and the structures were confirmed by ^1^H-NMR, UV–Vis, and MS spectroscopy.

**Figure 2:**
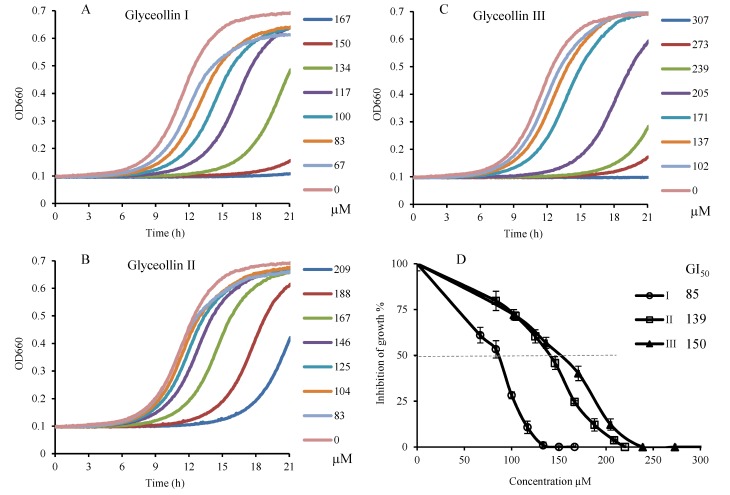
Influence of glyceollin I, II, and III on proliferation of yeast. These compounds were dissolved in methanol and added into YPD medium (5 μL compound: 100 μL medium) inoculated at 30 °C for 21 h. Growth curves of *S.* *cerevisiae* BY4742 at different concentrations of glyceollin I (**A**), II (**B**) and III (**C**) were monitored with a microplate reader by recording the optical density every 5 min at 660 nm. Relative inhibition of growth of yeast at different concentrations of glyceollins was calculated and the GI_50_ was the concentration that glyceollins inhibited 50% yeast cell growth. The growth inhibition was the observed after the 21 h of growth. Error bars represent standard error of the mean (SEM) within four replicates.

Glyceollins, one type of induced phytoalexins from soybean, were released in much higher concentration during plant growth in response to a number of stress factors such as wounding, freezing, ultraviolet light exposure, chemical and exposure to microorganisms [22]. Several studies had shown that their biological activities included antitumor, antiestrogenic, antibacterial, and antifungal effects [23]. To test the antiproliferation activity of glyceollins against budding yeast, approximately 2 × 10^4^ 2-day YPD (1% yeast extract/2% peptone/2% dextrose) cultured yeast cells in each well of a 96-well plate were treated with different concentrations of glyceollins, which were compared with methanol-treated controls (defined as 100% viability). The results showed that all three glyceollin isomers could inhibit the yeast proliferation (Figure 2), and 50% growth inhibition (GI_50_) of glyceollin I, II and III were 85, 139 and 150 µM respectively.

These GI_50_ values were consistent with a previous report showing that glyceollin I at 10 µM can reduce cell viability by 86% on MCF-7 breast cancer cells and by 90.32% on BG-1 ovarian cancer cells based on an assay of 1,000 cells per well [24]. Glyceollin I (^_^) has a GI_50_ value in the low- to mid-μM range for human breast, ovarian, and prostate cancer cell lines (<5,000 cells/well) [24]. In our assay, glyceollin I was more effective at reducing yeast viability than its isomers II and III. This result also agrees with a previous finding that the glyceollin isomer I had stronger bioactivity than isomers II and III on cancer cell line models [25,26]. According to a recent study, the mechanism of the inhibitory effects of glyceollins on platelet-derived growth factor (PDGF)-induced abnormal proliferation might be due to the influence on signal transduction events in the G_0_/G_1_-S interphase arrest. Glyceollins significantly reduce DNA synthesis in a dose-dependent manner without cytotoxicity and change the expression of cell cycle-regulatory proteins such as phosphorylated retinoblastoma protein (pRB), cyclin-dependent kinase (CDK)2 and cyclin D1, CDK inhibitor proteins p21^cip1^ and p27^kip1^, and tumor suppressors p53 [27,28]. Therefore, it is possible that the budding yeast antiproliferation assay could be used as a preliminary and rapid method for screening candidates with antifungal and anticancer activities, because the basic cellular processes among eukaryotes have a high degree of conservation [29].

### 2.2. Glyceollin I Extends Yeast CLS by a CR-Dependent Regime

**Figure 3:**
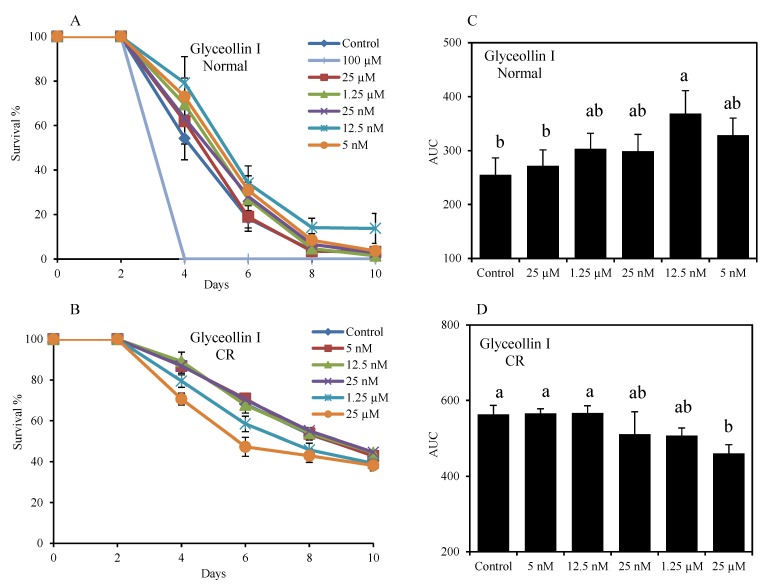
Glyceollin I as a CRM extends the budding yeast CLS. Glyceollin I was dissolved in methanol at different concentrations and added into the medium (5 μL compound: 1 mL medium) at day 2. (**A**) Survival curves of wild type BY4742 cultured in SD2D (SD medium containing 2% dextrose, normal condition) for 10 days, the control was added methanol without compound. (**B**) Survival curves of yeast in SD0.5D medium (0.5% dextrose, CR condition). Error bars represent SEM within 8 replicates. (**C**) Area under the survival curve (AUC) of different treatments under control and (**D**) CR condition; AUC represents the survival integral, the variance of AUC (mean + SEM, n = 8) between treatments was compared using the Duncan’s multiple range test at *p* < 0.05, different letters (a–b) show significant differences.

To test the anti-aging activity of glyceollins, they were dissolved in methanol and added into yeast culture at day 2 of the stationary phase, and the initial age-point (day 2) was defined to be 100% viability. As can be seen in Figure 3, under normal condition (2% glucose), glyceollin I in the range of 5 nM to 1.25 µM can extend life span (*p* < 0.05). The optimum concentration is at 12.5 nM, affording a maximum life span extension by 40% relative to the control. However, we found that glyceollin I could not extend CLS even at the optimal concentration (12.5 nM) under CR conditions (0.5% glucose) that could significantly extend yeast CLS. This suggests that glyceollin I mediates CLS extension and does not prevent life span extension through CR. Many natural small molecules such as caffeine, rapamycin, methionine sulfoximine, spermidine and lithocholic acid have been reported to extend yeast CLS, however, most of these compounds are not confirmed as CRM candidates [30,31]. A CRM should mimic the metabolic, hormonal, and physiological effects of CR under normal calorie intake. The CRM should activate stress response pathways observed under CR, provide protection against a variety of stressors, and produce CR-like effects on longevity with reduction of age-related diseases [32]. However, unlike glyceollin I, resveratrol and rapamycin, some natural products (such as lithocholic acid in the bile) do not operate as CRMs because they extend yeast CLS mainly under CR conditions [33]. This supports a unified hypothesis on how xenohormetic, hormetic and cytostatic selective forces within ecosystems drive the evolution of longevity regulation mechanisms in organisms across phyla [34].

**Figure 4:**
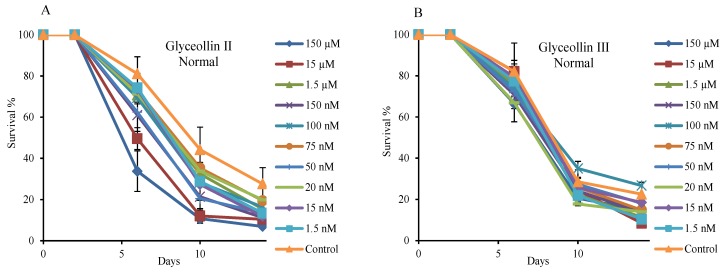
Glyceollin II (**A**) and III (**B**) do not extend yeast CLS. These compounds were dissolved in methanol at different concentrations and added into the medium (5 μL compound: 1 mL medium) at day 2. The control was methanol without compounds. Error bars represent SEM within eight replicates.

In comparison, glyceollin II and III had no effects on CLS (Figure 4) over a wide range of concentrations (5 nM to 150 µM). Glyceollin II reduced CLS at high doses. It is remarkable that the subtle structural variations of glyceollin I and II can result in such a dramatic difference in bioactivity. This indicates that structurally specific binding of the glyceollin I to yeast target is the critical event to exert bioactivity.

### 2.3. Hormetic Effect of Glyceollin I on Yeast Life Span

**Figure 5:**
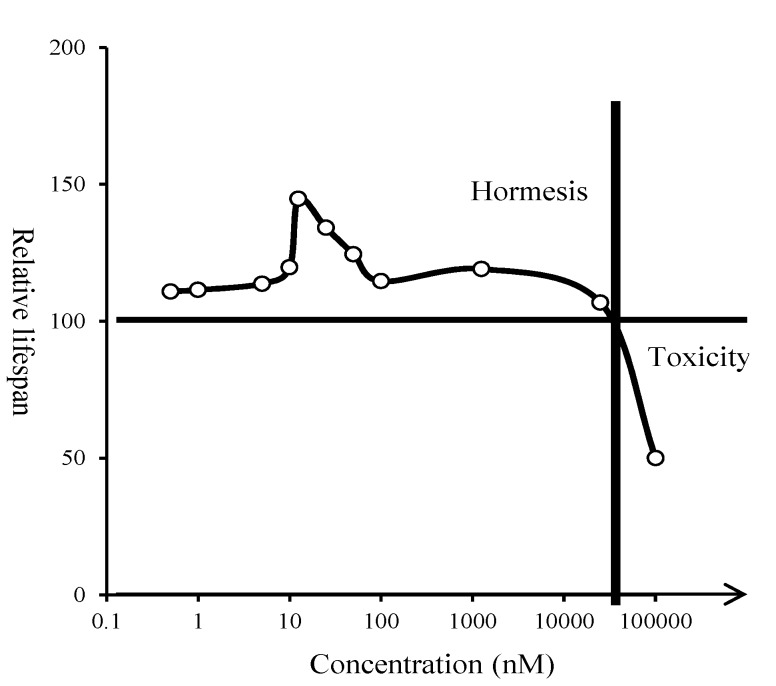
Dose-response curve of glyceollin I on yeast CLS with a hormetic effect. The testing concentrations were 0.5, 1, 5, 10, 12.5, 25, 50, 100, 1250, 25,000, 100,000 nM, respectively. Low doses resulted in life span extension, whereas higher doses resulted in life span reduction. The control with 100% life span was defined as adding methanol without glyceollin I, and the relative life span was based on AUC comparison of different doses relative to the control.

We measured the CLS extension activity of glyceollin I over a wide range of concentrations and determined a dose-response curve of glyceollin I on CLS showing a hormetic effect (Figure 5). At low doses (10 to 100 nM), glyceollin I induced yeast CLS extension. From 100 to 1.0 μM, there was negligible effect of glyceollin I on yeast CLS. Doses higher than 1.0 μM led to reduced CLS and toxicity (100 μM). Hormesis indicates that low concentrations of a toxin might have long-term beneficial consequences as a way of conditioning an organism toward enhanced stress responses. In our case, glyceollin I had the maximum CLS extension of only 40% relative to the control (Figure 5).

In fact, because the positive effects of a toxin occur at low doses, it has been reported that the benefits are typically only 30%–60% greater than controls [35]. Glyceollins are induced phytoalexins from soybean in response to stress factors. Therefore, we propose that glyceollin I would serve as stress-response hormesis agent to yeast and trigger specific physiological response of the fungus to extend their life span by a CRM regime.

## 3. Experimental

### 3.1. Materials

Food grade fungus, *R.* *oligosporus*, was bought from PT. Aneka Fermentasi Industri (Bandung, Indonesia), and black soybean (*Glycine max* (L.) Merr., China) was bought from a local supermarket in Singapore. HPLC grade acetone and methanol were obtained from Tedia Company (Fairfield, OH, USA). The 96-well polystyrene flat bottomed microplates were purchased from Fisher Scientific (Nunc, Rochester, NY, USA). Other solvents were of HPLC grade obtained from commercial sources. The wild-type strain *S. cerevisiae* BY4742 (MATα *his3*Δ1 *leu2*Δ0 *lys2*Δ0 *ura3*Δ0) was obtained from Thermo Scientific Open Biosystems (Huntsville, AL, USA). The culture of this yeast strain was aliquoted into 10 μL and stored at −80 °C. All L-amino acids were from GL Biochem (Shanghai, China), yeast nitrogen base (YNB) w/o amino acids and ammonium sulfate, peptone, agar, yeast extract were from Amresco (Solon, OH, USA). YPD Broth and other chemicals were from Sigma-Aldrich Chemical Company (St. Louis, MO, USA). MS spectra were acquired using a Finnigan/MAT LCQ ion trap mass spectrometer (San Jose, CA, USA) equipped with an electrospray ionization (ESI) source. The capillary temperature and spray voltage were maintained at 250 °C and 4.5 kV, respectively. ^1^H-NMR spectra were recorded in CDCl_3_ with a Bruker AC300 spectrometer (Karlsruhe, Germany) operating at 300 MHz. HPLC analysis was carried out on a Waters HPLC system (Milford, MA, USA) equipped with an Alliance 2659 separation module and a 2996 photodiode array (PDA) detector.

### 3.2. Isolation of Glyceollins

The black soybean seeds were germinated under fungal stress to induce glyceollins according to a previously described method [22]. The soybean seeds (2.0 kg) were germinated with *R. oligosporus* stress at room temperature (25 °C) in the dark for 3 days. The resulting germinated beans were homogenized in methanol, and then extracted three times on a shaking incubator at 200 rpm and room temperature for 6 h each time. The extracts were concentrated in a rotary evaporator at 50 °C. The concentrated residue was transferred to a silica gel column (35 × 6 cm, silica gel 60 (0.040−0.063 mm)) pre-equilibrated with hexane. The column was successively eluted with hexane and hexane/ethyl acetate (7:3) mixtures at a flow rate of 5 mL/min while numerous 100 mL fractions were collected. After HPLC analysis, the fractions containing the glyceollins were combined and the three isomers: glyceollin I, II, III (Figure 1A) were obtained and their identity confirmed by UV/Vis (Figure 1B), ESI-MS and ^1^H-NMR spectroscopy (data not shown). The detection wavelength during HPLC separation was set at 285 nm. The separation was accomplished on a Waters C18 column (Atlantis T3, 5 μm, 4.6 × 250 mm, Waters, Wexford, Ireland) with water (A), acetonitrile (B) and 2% acetic acid in water (C) as mobile phase. The column temperature was 30 °C. The injection volume was 20 µL. Solvent C composition was maintained at an isocratic 5% for 40 min. Solvent A and B gradient was as follows: 0–1 min, A 95%; 1–5 min, A from 95% to 50%; 8–36 min, A from 50% to 50%; 36–39 min, A from 50% to 90%; 39–40 min, A from 90% to 95%. The flow rate was 1.0 mL/min. 

### 3.3. Antiproliferation Assay

The yeast cells were prepared by streaking the strain BY4742 from frozen stocks onto YPD agar plates. After incubating the cells at 30 °C for 2 days or until colonies appeared, a single colony was selected and inoculated into a 1.0 mL YPD liquid medium in a 4-mL glass sample vial and cultured at 30 °C for 2 days in a flat incubator at 200 rpm. After 2 days of culturing (≈2 × 10^7^ cells/mL) in YPD media, 100 µL of the mixed culture was diluted with 10 mL YPD medium, then 100 µL of the diluted medium was added to each well containing 5 µL of glyceollins prepared in methanol (≈2 × 10^4^ cells/well). The cell population was monitored with a Synergy HT microplate reader (BioTek, Winooski, VT, USA) by recording the optical density (OD) every 5 min during 12–24 h at the wavelength of 660 nm.

### 3.4. Chronological Life Span Assay

CLS of yeast was measured according to the method described previously [36,37]. In brief, the 2-day YPD culture was diluted with autoclaved 18 MΩ Milli-Q grades water (1:10) and stored in refrigerator at 4 °C for at least 24 h. After one day incubation at 4 °C, 5 µL (≈1 × 10^4^ cells) of the diluted culture was transferred to a 1.0 mL of standard synthetic defined (SD) medium and maintained at 30 °C, 200 rpm for the entire experiment. After 2 days of culture in SD media, the cells reached stationary phase and the first age-point (defined as 100% survival) was ready to be taken. Five μL of methanol solution containing glyceollins was added into the medium at day 2. Subsequent age-points were taken every 2–4 days. For each age-point, 5.0 µL of the mixed culture was pipetted into each well of a 96-well microplate. One hundred µL of YPD medium was then added to each well. The cell population was monitored with the microplate reader by recording OD_660_ every 5 min during 12–24 h.

### 3.5. Data Analysis

The viability of the yeast was obtained according to previous method [21]. The raw data was exported to Excel (Microsoft, Redmond, WA, USA) and the OD curves were plotted as shown in Figure 2. From the growth curve, the viability of the yeast can be obtained through the following doubling time:

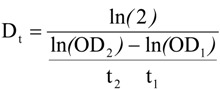

where OD_1_ and OD_2_ represent successive OD measurements, and t_1_ and t_2_ are the time between measurements. The average doubling times (Dt_n_), defined as the average of the five lowest (except the first one) doubling times only between OD values of 0.2 to 0.5, is the doubling time for that well. The lag time was calculated as follows:

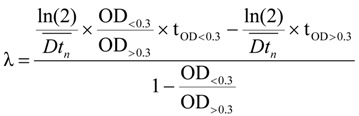

where OD_>0.3_ and OD_<0.3_ represent the OD value first measured to be greater than 0.3001 or less than 0.3001 (0.3001 is to avoid the case that OD is 0.300), and t_OD>0.3_ and t_OD<0.3_ are the time corresponding to the intersection of the maximal slope of the ln curve with the x-axis [38]. An easy way to determine OD_<0.3_ and OD_>0.3_ is to use the Excel function “SMALL” and “LARGER”. Next, the function “MATCH” was used to determine the relative position (order) of OD_<0.3_ and OD_>0.3_ in the running time range of the column. Finally, the function “INDEX” was used to return t_OD>0.3_ and t_OD<0.3_ values of the cell at the intersection of the row of order and the column of time, in the running time range (0–24 h). It took different length of time for each well to reach an OD of 0.3 between the initial age-point and each subsequent age-point. For each age-point, the time shift was calculated as follows:

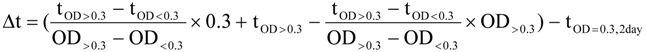

where t_OD = 0.3,2day_ is the time that OD value of day 2 age-point reaches 0.3 in the outgrowth curves. The initial age-point (day 2) is defined to be 100% viability and the relative survival percent of each successive age-point can be calculated as follows:

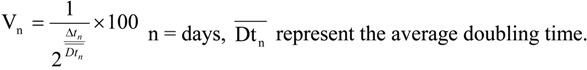


The survival integral (SI) for each well is defined as the area under the survival curves (AUC) and can be estimated by the formula:

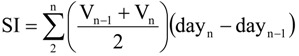

where day_n_ is the age point, such as day 2, 4, 6, 8, 10 [39]. The analysis of variance for each set of biological replicates was carried out with the SAS statistical program version 9.00 (SAS Institute Inc., Cary, NC, USA), and differences between the means of SI for treatments were determined by Duncan’s multiple range test at *p* < 0.05.

## 4. Conclusions

In conclusion, we have presented the anti-proliferation and anti-aging activities of the induced phytoalexins glyceollin I, II and III from soybean in the budding yeast model. The three glyceollin isomers showed strong anti-proliferation activity at μM doses and glyceollin I had lower GI_50_ than the other isomers. Interestingly, it was found that glyceollin I could extend yeast life span at nM doses. Furthermore, the longevity effect was a CR-dependent. However, glyceollin II and III did not have hormetic effects on yeast life span. Glyceollin I has many bioactivities including anti-bacterial, anti-nematode, anti-fungal, anti-estrogenic and anti-cancer, anti-oxidant, anti-inflammatory, insulin sensitivity enhancing, and vascular contraction attenuation properties [23]. Our discovery adds glyceollin I to the list as a promising CRM candidate. The biological mechanism of this glyceollin I bioactivity remains to be elucidated so that we can rationally alter the structures of glyceollins to improve the CRM effects.
